# P-2037. Overutilization of BAL PCR BioFire panel in Lung Transplant Recipients: a Perspective from Diagnostic Stewardship

**DOI:** 10.1093/ofid/ofaf695.2201

**Published:** 2026-01-11

**Authors:** Allison Giuffre, Marin Schweizer, Brandon Leding, Lindsay Taylor, Chris Saddler, Derrick Chen, Mike Scolarici

**Affiliations:** University of Chicago , Chicago, IL; William S. Middleton VA Hospital, madison, Wisconsin; University of Wisconsin-Madison, Madison, Wisconsin; University of Wisconsin School of Medicine and Public Health, Madison, Wisconsin; University of Wisconsin-Madison, Madison, Wisconsin; University of Wisconsin School of Medicine and Public Health, Madison, Wisconsin; University of Wisconsin-Madison, Madison, Wisconsin

## Abstract

**Background:**

Lung transplant (LTx) recipients require lifelong immunosuppression and monitoring for respiratory infections and graft rejection. The American Society of Transplantation recommends molecular tests when evaluating pneumonia, and in 2023 our lab implemented the BioFire FilmArray Pneumonia panel, a multiplex PCR assay designed for lower respiratory tract specimens including bronchoalveolar lavage (BAL) fluid. This test does not distinguish pathogenic from colonizing organisms and may lead to excessive antimicrobial use or inappropriate reduction in immunosuppression.

**Figure:**
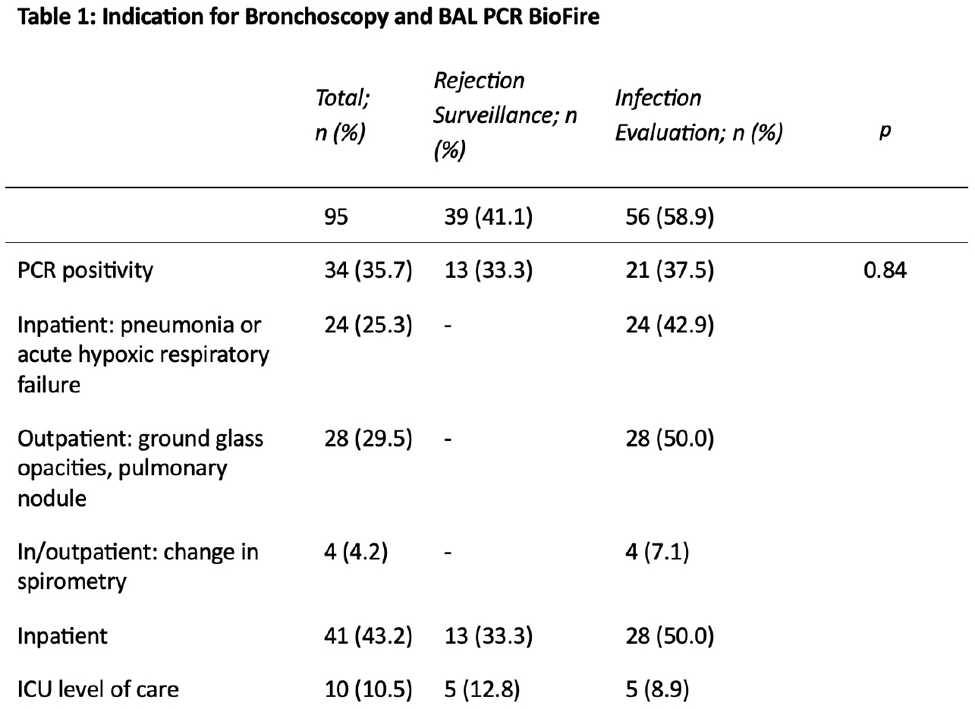

**Figure:**
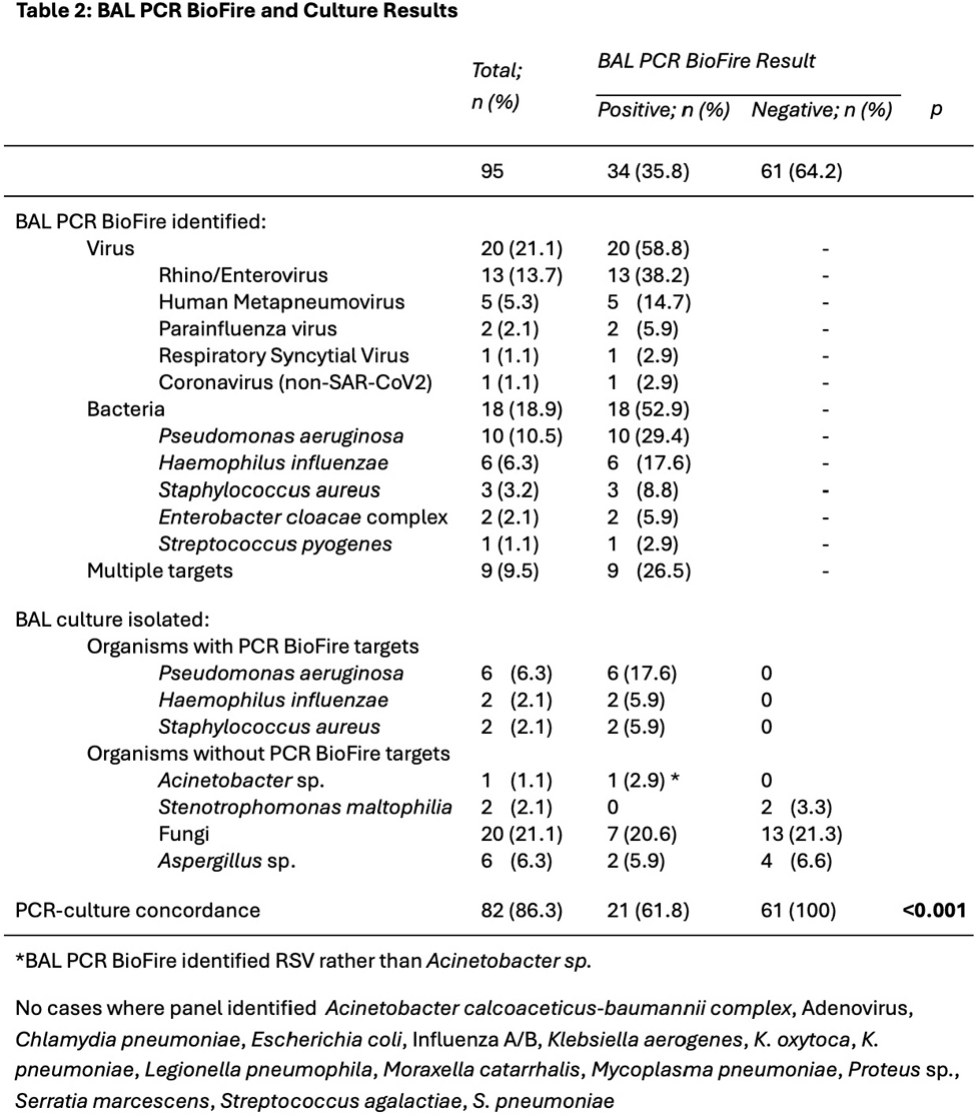

**Methods:**

This retrospective cohort included adult LTx recipients from February 2023 to October 2023 with simultaneous BAL bacterial culture and PCR BioFire. PCR positivity was compared by treatment team reported bronchoscopy indication: rejection surveillance or infection evaluation. PCR-culture concordance was defined as both methods identifying the same PCR targeted bacterial pathogens. Pre/post-test antimicrobials and mycophenolate dosing for immunosuppression were compared. R version 4.4.1 was used for statistical analyses: bivariate logistic regression to assess factors associated with PCR positivity and chi-square to evaluate categorical variables.

**Figure:**
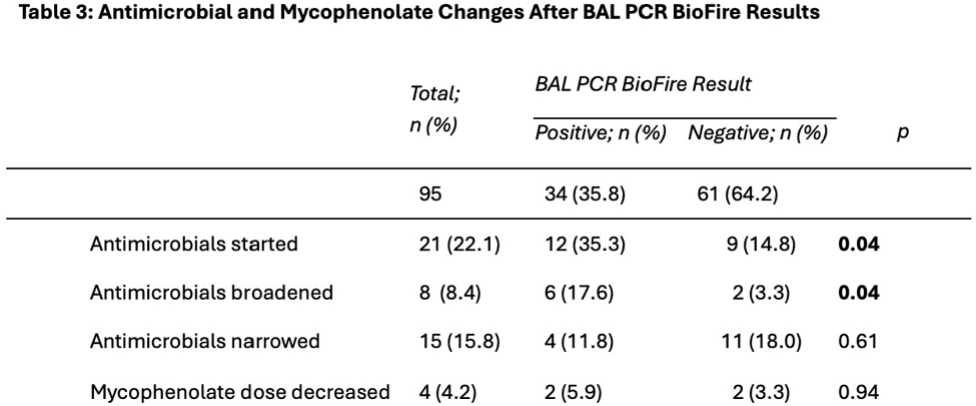

**Figure:**
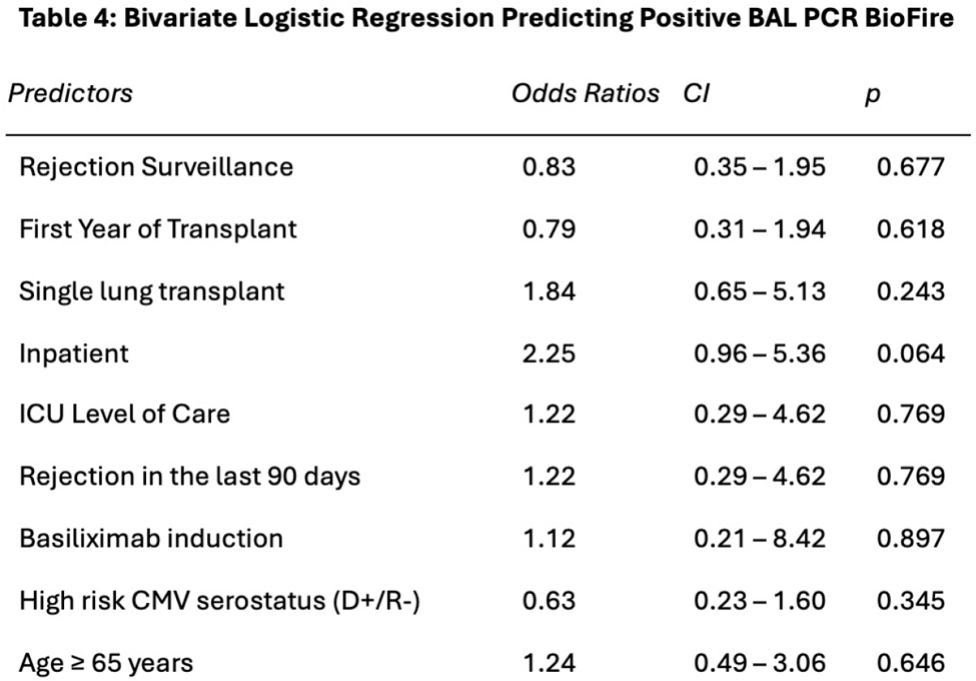

**Results:**

We included 95 LTx recipients [mean age 58 years, 20% single LTx, 94% basaliximab induction immunosuppression, 85% LTx in 2015 or later, 10% diagnosed with rejection in prior 90 days]. The PCR panel was collected for rejection surveillance in 41% and infection evaluation in 59%, and there was no difference in PCR positivity (33.3% v 37.5%; p=0.84) (Table 1). PCR-culture concordance was high (Table 2). Antimicrobials were more frequently started (35.3% v 14.8%; p=0.04) and broadened (17.6% v 3.3%; p=0.04) after a positive than negative PCR; there were no differences in narrowing antimicrobials or decreasing mycophenolate dosing (Table 3). There were no significant factors associated with PCR positivity (Table 4).

**Conclusion:**

While LTx recipients are at high risk for pulmonary infections, the PCR panel was frequently positive regardless of the pre-test concern of infection. The PCR panel should not be routinely ordered during rejection surveillance in the absence of clinical concern for infection.

**Disclosures:**

All Authors: No reported disclosures

